# Development and Validation of the Ethnic Moral Disengagement Scale

**DOI:** 10.3389/fpsyg.2021.756350

**Published:** 2022-01-17

**Authors:** Maria Grazia Lo Cricchio, Federica Stefanelli, Benedetta E. Palladino, Marinella Paciello, Ersilia Menesini

**Affiliations:** ^1^Department of Humanities (DiSU), University of Basilicata, Potenza, Italy; ^2^Department of Education, Languages, Intercultures, Literature and Psychology, University of Florence, Florence, Italy; ^3^Uninettuno Telematic International University, Rome, Italy

**Keywords:** moral disengagement, ethnicity, ethnic bullying, ethnic cyberbullying, scale development

## Abstract

Research has underlined that moral disengagement processes, by which people switch off their moral values and act aggressively without experiencing guilt, are highly connected with contextual factors. However, research on situational variations in moral disengagement is limited, especially considering the associations with characteristics such as the ethnic origin of potential victims. The general aim of the present study was to develop a brief, specific measure of ethnic moral disengagement able to catch individual justification used in the case of ethnic bullying and cyberbullying, and test its validity and reliability. An eight items scale was developed and administered in study 1, in a sample of 961students attending several Italian high schools (53.5% female; Mage 15 years). Considering the results of the CFA, we modified one of the items and the scale was administered again, in a second sample of 1,229 students (49.9% female; Mage 15.62 years) in study 2. A one-factor model of ethnic moral disengagement fit the data well and internal consistency showed to be good. As an additional step, we found that the model was invariant across Italian adolescents and youths with a different ethnic or culture of origin (having at least one parent born abroad) strengthened our confidence regarding the factorial integrity of the scale. Last, the scale showed to be positively associated with ethnic bullying and cyberbullying. Generally, findings suggested that the Ethnic Moral Disengagement scale can be a useful tool for those interested in measuring moral disengagement and evaluating how it impacts bullying and cyberbullying of minority groups.

## Introduction

Globalization has increased the movement of many people from one country to another, thus promoting processes of migration (Fandrem et al., 2012). In this context, it must be considered as pivotal to gaining a better understanding of the factors which encourage positive intercultural relationships, thus reducing intolerance and discriminatory behaviors. Since mechanisms of Moral Disengagement (MD) are particularly informative with respect to discriminatory and racial behaviors (Faulkner and Bliuc, 2016; D’Errico and Paciello, 2018), the present study is aimed at contributing to the literature concerning this issue, by presenting a new scale aimed at measuring Ethnic MD (EMD).

As the moral self develops, individuals adopt standards of right and wrong that will guide their moral conduct. According to theory of moral agency Bandura’s (1991, 2016), moral self-regulation processes promote ethical conduct, and prevent unethical behaviors, by means of proactive or inhibitive mechanisms. The proactive process supports ethical conduct by regulating how behavior fits with personal and social standards, while the inhibitive process impedes negative actions by seeing them as ethically and socially punishable. So, when individuals engage in moral conduct, they may feel guilty or proud, depending on these processes of self-monitoring and judgment. However, moral self-regulation does not always work in a consistent manner (Bandura, 2015), and, under specific circumstances, certain cognitive practices lead an individual to disengage from their own moral principles, and to behave unfairly. These processes have been proposed as the mechanisms of MD, which work by restructuring the four dimensions of behavior representation, or *loci* of cognitive restructuring: behavior, agency, consequences, and victims (Bandura, 1991).

The *behavior locus* refers to the maneuvers focused on changing the meaning of harmful conducts, and it includes moral justification (the cognitive redefinition of negative behaviors as respectable), euphemistic labelling (the use of language that cognitively masks blameworthy actions as less harmful), and advantageous comparison (comparing negative behaviors with more unacceptable behaviors, thus making them appear better or less severe). The *locus of agency* refers to mechanisms aimed at avoiding personal responsibility, and it includes displacement of responsibility (viewing one’s own actions as the result of social pressures and thus not under one’s personal responsibility), and diffusion of responsibility (when duty is shared with others, thus reducing personal responsibility and motivation to action). The *locus of consequences* refers to processes aimed at altering one’s perception of the effects of their own behavior, by disregarding or distorting its results (avoiding or cognitively minimizing the harm caused by bad acts). Finally, the *victims’ locus* of redefinition refers to attempts to displace responsibility onto the victim *via* mechanisms of dehumanization (depriving victims of human qualities or attributing animalistic characteristics to them) and by attribution of blame (attributing victims the fault of injurious or provocative conducts). By using these MD processes, people can concretely switch off their moral values and act wickedly and aggressively without experiencing shame, guilt, or blameworthiness (Bandura, 1991; Paciello et al., 2008). Despite the different dimensions and *loci*, Bandura (1991) suggested that all mechanisms of MD are part of a single construct, and that MD maneuvers are only diverse ways to pursue the same and unique aim of decreasing guilt for one’s detrimental conduct. This theoretical idea has been confirmed by different studies in which MD was evaluated with diverse scales, including items measuring each mechanism on MD. Findings have shown a single factor structure for this construct, when it was measured by one item for each dimension (8-item scales; Boardley and Kavussanu, 2008; Lucidi et al., 2008; Moore et al., 2012) or a common, second order, latent variable when more items were included (Bandura et al., 1996, 2001; Caprara et al., 1996; Pelton et al., 2004).

Research has shown that MD is strictly linked to aggressive behavior, including traditional bullying in schools (e.g., Bandura et al., 2001; Gini et al., 2014; Kowalski et al., 2014), and cyberbullying (e.g., Lo Cricchio et al., 2021). In particular, literature underlined that bullying perpetrators are more likely to score higher in MD than those not involved in bullying (Menesini et al., 2003; Caravita et al., 2012; Thornberg and Jungert, 2013). Moreover, bystanders with higher levels of MD are less likely to defend the victims when witnessing episodes of bullying (Gini, 2006; Obermann, 2011; Caravita et al., 2012; Thornberg and Jungert, 2014).

MD must be considered a product of the reciprocal interaction between individual and social and/or situational factors: it is not a trait or a disposition, but a process that can be selectively activated under different conditions (Bandura, 1999, 2016). Nevertheless, in a recent meta-analysis concerning the association between MD and bullying, Killer et al. (2019) concluded that there is a lack of investigation of the broader impact of these situational contexts, and underlined the need for further research into how MD and contextual variables may interact and explain aggressive and bullying behavior. Studies suggest, in fact, that the associations between MD and bullying can be affected by specific factors, such as the characteristics of the victims and the relational context (Thornberg et al., 2020). As in the case of discriminative aggression, it seems plausible to expect that the ethnic origin of potential victims may play a role.

Previous research on anti-immigrant attitudes and prejudicial bullying behaviors indicates that MD may be important to explain why some youths perpetrate aggression toward their peers with different ethnic or cultural backgrounds. For example, it has been suggested that the likelihood of harassing immigrants is increased by negative attitudes and preconceptions toward them, and by having strong beliefs that immigrants deserve any negative treatment they receive (Bayram Özdemir et al., 2020).

Within the bullying context, research suggested that bullies’ perception of victims as different because of their immigration status can increase the risk of harassment (Caravita et al., 2019). In fact, the more immigrants are perceived as different and not fitting peer group norms and characteristics, the more this can cause their peers to mark them as dangerous or deviant, eliciting MD processes in which bullies justify themselves as acting to protect their group from the aberrant outsiders. Furthermore, prejudices and stereotypes might activate specific MD processes such as dehumanization of the victim, through which bullies become more compelled and disposed toward acting cruelly and harshly toward victims who are ethnically diverse (Webster and Saucier, 2015; Bandura, 2016).

However, one of the main limits of the available knowledge on these issues is related to how MD has been measured. Despite how Bandura (1977) claimed that the closer the cognitions are to the actions, the stronger the explicative power of the measure, in the majority of studies, MD has been assessed as a general disposition by using items such as those of the traditional measure of Bandura et al. (1996). These measures usually ask to express personal opinions concerning negative behaviors toward people, without considering the contextual factors, such as who these individuals are, and, in particular, how the different ethnic origins of potential victims can influence adolescents’ cognition and behavior.

Even if some scholars have developed more specific measures of MD, such as for cyber (Paciello et al., 2020), civil (Caprara et al., 2009), organizational (Moore et al., 2012), and sports contexts (Boardley and Kavussanu, 2008), to the best of our knowledge, only one study has proposed to evaluate MD in interethnic relations with *ad hoc* measures (Caravita et al., 2019). In particular, Caravita et al. (2019) used vignettes in which the target of bullying is a non-immigrant vs an immigrant new classmate. For each scenario, participants must answer sixteen items, for a total of thirty-two items. The use of vignettes can have potential strengths, but also some limitations. The main strength is related to the possibility of having direct examples of ethnic bullying episodes, but at the same time, the specificity of the situations may limit the reported reactions to those contexts, without providing a more general perception of ethnic bullying. Besides, the vignettes may be more appropriate for younger students, whereas a general brief scale may be more suitable for older students and, in general for school administration purposes, where limited time is often requested by teachers. Therefore, a brief, valid, and reliable measure of EMD would be an added value in the research field and serve scholars who wish to incorporate a specific MD measure in a multivariate investigation.

### The Present Study

To sum it up, the literature indicates that MD mechanisms are highly connected with contexts and to interethnic relationships and dynamics. However, research on situational variations in MD is limited, especially considering characteristics such as the ethnic background of potential victims. Hence, the general aim of the present study was to address this gap in knowledge by developing a specific and short measure of EMD, able to catch individual justifications and interpersonal mechanisms used in cases of ethnic bullying and victimization. In developing the new scale, we followed recommendations for constructing and revising scales (e.g., Smith et al., 2000). Generally, (a) the factorial dimensionality of the instrument must be examined by factor analyses (CFA); (b) factor must demonstrate standards of reliability; (c) the newly developed instrument must be administered to a different sample from the one used when the scale was originally constructed; (d) the factor structure and the reliability of its factor must be confirmed; and (e) the newly developed scale must be validated. We followed these guiding standards in carrying out two studies. In particular, study 1 aims at developing and testing the factor structure of the scale, whereas study 2 aims at evaluating its validity, reliability and structural invariance.

## Study 1

The first study aims at developing and evaluating the factor structure of the scale of EMD in a sample of students attending Italian high schools.

### Method

#### Participants and Procedure

Participants were 1,311 students nested in 58 classes of 13 Secondary Schools in Italy, all of which attended Lyceum, Technical or Vocational high schools (grade 9). Before questionnaire administration, informed consent, consisting of initial approval by the School Principal and the class council, was requested. Once permission was gained from schools, informative letters were sent to all students and to their parents, explaining the study aims and requesting the parents’ consent for their child’s participation. 1,153 students were present at school on the day of the data collection but data were retrieved only by 961 students because 192 did not have parental authorization. Of the 961 students who filled the questionnaire, 437 (46,5% of the whole sample) were male, while 503 (53,5% of the whole sample) were female (21 students did not answer the question about gender). Students’ mean age was 15 years old with a standard deviation of 0.60 (MAXage = 18 years old; MINage = 11 years old). Most of the participants were Italian, having both parents born in Italy (71.1% of the whole sample). 278 students (28.9% of the whole sample) had a different ethnic or culture of origin, having at least one parent born abroad. The students with an immigrant background came from various countries of the world, such as China (4.2 %), Albania (2.7 %), Morocco (1.3 %), Romania (1.1 %), and other countries (19.6 %).

Before collecting data, institutional ethical committee approval was obtained for the study procedure. The schools that took part in the research were recruited for a voluntary census. Specifically, the call for participation was extended to all the high schools in several Italian provinces. The study survey was administered in January 2020 by trained assistants during school hours. Of the 961 high school students who participated in the study, 509 filled the paper version of the questionnaire, while 452 filled the online one, using school computers.

#### Measure

##### Ethnic Moral Disengagement Scale

Prior to all steps, we developed an initial set of eight items to measure MD related to ethnic minority potential victims. The general references for this aim were: (a) the theory of MD proposed by Bandura (1991); and (b) the use of existing items concerning MD, such as those of the Online MD (Paciello et al., 2020). In developing the scale, each MD process was represented with one item. This initial pool of eight items was reviewed by a professional with research expertise relating to the fields of ethnicity and MD. Items were adjusted following their feedback, resulting in a final 8-item scale that is presented in Table 1. The initial set of eight items to measure MD related to ethnic minority potential victims was administered to participants. Each item was evaluated along a 5-point scale (strongly disagree, disagree, quite agree, agree, and totally agree).

**TABLE 1:** EMD scale items and mechanisms of moral disengagement.

EMD items	MD mechanisms
1. Bullying children of different ethnicities or origins is just a way to spend time with friends	Euphemistic labeling
2. There is no reason why boys/girls of different ethnicities or origins get offended when they are teased, because this is still a way to pay attention to them.	Disregarding/Distorting consequences
3. If any boy/girl of different ethnicity or origin is treated badly by others, it is because he/she is the first to behave badly toward Italians.	Attribution of blame
4. It is right to exclude boys/girls of different ethnicity or origin to defend our culture	Moral justification
5. People of different ethnicities or origins who are mistreated usually deserve it because they are like beasts	Dehumanization
6. It is not serious to insult someone of a different ethnicity or origin since beating them would be even worse	Advantageous comparison
7. If most parents provide a bad example, it is not the children’s fault if they denigrate those of a different ethnicity or origin.	Displacement of responsibility
8. Young people should not be blamed for insulting those of a different ethnicity or origin since most Italians do the same	Diffusion of responsibility

#### Analytic Plan

As a preliminary step, we looked at missing values in the matrix. Thus, we tested if missing data occurred completely at random (MCAR) using Little’s test analysis. Little (1998) has proposed a statistical test of the MCAR assumption, which is a chi-square test. Significant chi-square values indicate that the data are not MCAR.

After controlling for MCAR assumption, firstly, we explored items’ distributions and correlations by performing a descriptive analysis. Not all the items included in the EMD scale presented symmetric distribution. Thus, we proceeded to examine the factorial structure of the EMD scale, using robust methods for the estimation of the parameters. In particular, following Bandura’s theoretical model, and literature indications, we tested the predicted one-factorial structure of the EMD scale, performing a Confirmatory Factor Analysis using the R packages Lavaan (Rosseel, 2012). Specifically, we ran a monofactorial model (latent factor: EMD, items: 1, 2, 3, 4, 5, 6, 7, and 8). The model was evaluated according to the following indices: the chi-square (χ2) statistic, the root-mean-squared error of approximation (RMSEA), the comparative fit index (CFI), the Tucker-Lewis Index (TLI), and the standardized root mean squared residual (SRMR). Recommended cut-off points for these measures are 0.08 (Brown and Cudek, 1993) or 0.06 (Hu and Bentler, 1998) for RMSEA, 0.90 or 0.95 for CFI and TLI (Bollen, 1989) and 0.08 or 0.05 for SRMSR (Hu and Bentler, 1998). The cut-off used for the factor loading was 0.30 (Muthén and Muthén, 2007). Finally, to evaluate the reliability of the scales, we analyzed the internal consistency of the dimension by means of Cronbach’s alpha. The analyses were conducted *via* R Studio (R Studio Team, 2020).

## Results

### Descriptive Statistics

Descriptive statistics and bivariate associations between the eight items of the EMD scale are reported in Table 2.

**TABLE 2:** Descriptive statistics of the items of the EMD scale (study 1).

	N	Mean	SD	Skewness	Kurtosis	2	3	4	5	6	7	8
1. Item 1	895	1.33	0.72	2.54	6.77	0.36**	0.26**	0.39**	0.34**	0.31**	0.07**	0.29**
2. Item 2	891	1.62	1.0	1.69	2.19	–	0.35**	0.35**	0.36**	0.35**	0.19**	0.32**
3. Item 3	895	1.80	0.96	1.20	1.01	–	–	0.40**	0.36**	0.38**	0.19**	0.31**
4. Item 4	889	1.37	0.76	2.32	5.20	–	–	–	0.61**	0.58**	0.11**	0.38**
5. Item 5	892	1.31	0.72	2.70	7.53	–	–	–	–	0.50**	0.13**	0.37**
6. Item 6	891	1.49	0.93	2.15	4.24	–	–	–	–	–	0.14**	0.47**
7. Item 7	887	2.50	1.25	0.44	−0.78	–	–	–	–	–	–	−26**
8. Item 8	890	1.69	1.07	1.59	1.76	–	–	–	–	–	–	–

**p < 0.01.

As Table 2 shows, not all the items included in the EMDS presented symmetric distribution. Indeed, the items Skewness indexes range from 0.44 to 2.70, while the Kurtosis indexes range from −0.78 to 7.53. All the items included in the scale are correlated with each other, but not too strongly.

### Factorial Structure of the Ethnic Moral Disengagement Scale

Our data were missing completely at random as indicated by the non-significant Little’s (1998) MCAR test [χ2(81) = 96.75, *p* = 0.111]. Thus, we proceeded by using the full information maximum likelihood approach (FIML) (Enders and Bandalos, 2001) for the estimation of missing data in our matrix.

The model fit indices were all satisfactory except for Chi Squared p, which is especially sensitive to sample size [χ2(20) = 71.94, *p* < 0.001]. Specifically, RMSEA, SRMR, TLI, and CFI had optimal values in the monofactorial solution (RMSEA = 0.075; SRMR = 0.041; TLI = 0.921; CFI = 0.944).

**FIGURE 1:**
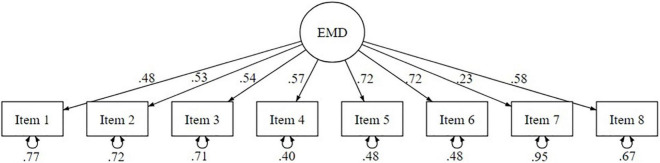
Graphical representation of the Ethnic Moral Disengagement monofactorial model (study 1).

The standardized estimates are reported in Figure 1. Not all factor loadings were satisfactory. Indeed, while the factors loadings of the items 1, 2, 3, 4, 5, 6, and 8 were ranged from βItem1 = 0.48 to βItem5-6 = 0.72, the factor loading for the item 7 was βItem7 = 0.23 (SE = 0.139; *p* < 0.001). We used Cronbach’s alpha coefficients to calculate the scale’s internal consistency. EMD scale showed decent reliability [α = 0.77; 95% CI (0.75–0.80)].

## Study 2

The first study revealed some limitations of the scale for the assessment of EMD. In particular, Item 7 was not sufficiently adequate for measuring the latent factor EMD. Therefore, we reformulated it and we administered the EMD scale in a different, independent, sample to test its psychometric characteristics. We examined its factorial structure, internal consistency, and ethnic measurement invariance. We also evaluated the discriminant validity of the scale. To do so, we examined whether the score on its factor was associated with ethnic bullying and cyberbullying behaviors. Specifically, we expected that EMD to be positively correlated with both traditional and virtual forms of prejudicial ethnic bullying behaviors.

### Participants and Procedure

Before collecting data, institutional ethical committee approval was obtained for the study procedure. We recruited a new larger sample composed of 1,636 students nested in 77 classes of 11 Secondary Schools in Italy. All of the participants attended Lyceum, Technical or Vocational high school (grades 9, or 10). Before questionnaire administration, informed consent, consisting of initial approval by the School Principal and the class council, was requested. Once permission was gained from schools, informative letters were sent to all students and to their parents, explaining the study aims, and requesting the parents’ consent for their child’s participation. The study survey was administered from February to March 2021. During that period, due to COVID-19 pandemic, schools were closed and students studying from home, so we had them fill the questionnaire online, under the supervision of trained assistants.

On the day of the data collection, 203 students were not following online classes. Of the remaining 1,433 high school students, 67 did not give their authorization for participation in the study, and 136 did not send their questionnaire answers, because of problems with their internet connections. Moreover, we decided not to consider the questionnaire responses provided by one student because he was not an adolescent (26 years old). Thus, overall, 1,229 students filled the questionnaire (49.9% female, and 50.1% male). Students’ mean age was 15.62 years old (SD = 0.72; MAXage = 18 years old; MINage = 14 years old). While most of the participants were Italian, with both parents born in Italy, 275 students had an immigrant background, having at least one parent born abroad (416 students did not answer either of the questions about their parents’ nationality). Specifically, not considering the missing data, 10.4% of the students’ mothers come from other European countries, mostly from Albania (4.1%) and Romania (2.6%), while 9.1% come from non-European countries, mostly from Morocco (2%) and China (1.5%). On the other hand, 8.1% of the students’ fathers come from other European countries, mostly from Albania (4.1%) and Romania (2%), while 10% were from non-European countries, mostly from Morocco (2.1%) and China (1.4%). In the following paragraphs, the label “with a different ethnic/culture of origin” will refer to students of whom at least one parent was born abroad. On the contrary, the label “Italian students” will refer to youths whose parents were both born in Italy.

### Measure

#### Ethnic Moral Disengagement Scale

We administered the same 8-items scale of study 1, with only the modified version of item 7. In fact, since this item resulted as less adequate for measuring the latent factor EMD, we reformulated it with the help of an expert in the field. Maybe, the original item was not sufficiently unequivocal. Therefore, we changed it to clarify its meaning and in order to simplify its understanding. In particular, we reformed the item from “If most parents provide a bad example, it is not the children’s fault if they denigrate those of a different ethnicity or origin” to “It is not the child’s fault if they exclude those of a different ethnicity/origin, if most parents set a poor example.”

#### Ethnic Bullying

We administered a modified version of the Florence Bullying Scale (Palladino et al., 2016, 2020) that ask how often, in the previous couple of months, students behaved like bullies, attacking other students with an immigrant background physically, verbally, and or indirectly (i.e., “I beat someone up because of his/her culture or country of origin”). A definition of bullying introduced the scale, consisting of four items. Each item was evaluated along a 5-point scale from “never” to “several times a week.” Within our data, the scale presents acceptable internal consistency [α = 0.89, 95% CI (0.88–0.90)].

#### Ethnic Cyberbullying

We used a modified version of the Florence Bullying Scale (Palladino et al., 2016, 2020) that asks how often in the previous couple of months students behaved like cyber bullies, excluding other students with a different ethnic or culture of origin from the online group, and/or taking their personal information to reuse later, and/or sending embarrassing photo or videos, and/or sending threats and insults on the Internet (i.e., “In the last 2 or 3 months, how often have you sent threats and insults on the internet to someone because of his/her culture or country of origin?). A definition of cyberbullying introduced the scale, consisting of four items. Each item was evaluated along a 5-point scale from “never” to “several times a week.” Within our data, the scale presents acceptable internal consistency [α = 0.81, 95% CI (0.79–0.82)].

### Analytic Plan

As a preliminary step, we checked if missing values occurred completely at random (MCAR) using Little’s test analysis (Little, 1998). Since our data were missing completely at random, we proceeded using the full information maximum likelihood approach (FIML) (Enders and Bandalos, 2001) for their estimation. After controlling for the MCAR assumption, firstly, we explored the distribution and the correlations of the items included in the EMD scale performing descriptive analysis. Not all the items presented a symmetric distribution, thus, we proceed to test our model using the robust method.

To examine the hypothesized one-factor structure of the EMD scale, we performed a Confirmatory Factor Analysis using the R packages Lavaan (Rosseel, 2012) testing a monofactorial model. The model was evaluated according to the same indexes and the same recommended cut-off used in the first study: are 0.08 (Brown and Cudek, 1993) or 0.06 (Hu and Bentler, 1998) for RMSEA, 0.90 or 0.95 for CFI and TLI (Bollen, 1989) and 0.08 or 0.05 for SRMSR (Hu and Bentler, 1998). We also evaluate the reliability of the scales, analyzing the internal consistency by means of Cronbach’s alpha.

As a second step, we tested for measurement invariance to verify whether the instrument has the same psychometric properties across the majority (i.e., Italians) and the minority (i.e., students with different ethnic or culture of origin). We followed the procedures described by Meredith (1993), and Widaman and Reise (1997). The sequence of invariance testing starts from the configural invariance, which involves running a model in which all parameters are estimated freely (A configural –1st level). At this step of measurement invariance, only the similarity across groups of the overall parameters’ pattern is evaluated. This provides indications about the ability of the original model to fit the data in each group (here, Italian students and students with different ethnic or culture of origin) without invariance constraints. The invariance measure proceeds step by step, comparing increasingly restricted models. The 2nd level of invariance involves constraining factor loadings over the groups as invariants (B metric–2nd level). The third level of invariance involves a stricter model in which both factorial loadings and intercepts are constrained across groups (C scalar–3rd level). The fourth level of invariance is tested at residual variance invariance (D strict—4th level). Finally, the fifth level of invariance involves a model in which both factor loadings, intercepts, residual variance, and factor variance are constrained to be equal across groups (E factor variance—5th level). To summarize, each level of invariance involves an even more restricted model. Each one of these models, from the least (1st level) to the most restrictive one (5th level), is nested in the original model. Moreover, we tested a very strict model, in which the means equality was also imposed across the two different ethnic groups (F latent mean 6th level) (Vandenberg and Lance, 2000).

The goal of each level of the measurement invariance is to make the model fit, not to worsens it by constraining parameters equally across groups. Thus, at each level of the analysis, we tested whether subtracting parameters worsened the model fit by controlling the change in the fit indices RMSEA and CFI. It has been suggested (Chen, 2007) that support for invariance across groups requires that at each step of the analysis the CFI is not worse more than—0.01 across models and RMSEA is no worse than 0.015 across models. We also considered the Akaike Information criterion (AIC) and Bayesian Information Criterion (BIC) in testing for the evidence of invariance (Vrieze, 2012): lower AIC and BIC value indicates a better trade-off between fit and complexity.

Finally, we evaluated the discriminant validity of the scale, checking whether the EMD scale was correlated with ethnic bullying and ethnic cyberbullying behaviors. The analyses were conducted by R Studio (R Studio Team, 2020).

## Results

### Descriptive Statistics

Descriptive statistics and bivariate associations between the eight items of the EMD scale are reported in Table 3.

**TABLE 3:** Descriptive statistics of the items of the EMD scale (study 2).

	N	Mean	SD	Skewness	Kurtosis	2	3	4	5	6	7	8
1. Item 1	1,172	1.29	0.72	2.61	6.75	0.41**	0.38**	0.46**	0.48**	0.44**	0.25**	0.36**
2. Item 2	1,172	1.47	0.92	2.06	3.66	–	0.39**	0.45**	0.49**	0.43**	0.28**	0.4**
3. Item 3	1,171	1.67	0.90	1.44	1.98	–	–	0.53**	0.49**	0.45**	0.31**	0.39**
4. Item 4	1,171	1.36	0.77	2.26	4.84	–	–	–	0.68**	0.62**	0.27**	0.49**
5. Item 5	1,171	0.130	0.75	2.65	6.73	–	–	–	–	0.62**	0.29**	0.05**
6. Item 6	1,171	1.39	0.82	2.35	5.35	–	–	–	–	–	0.34**	0.46**
7. Item 7	1,171	1.98	1.04	0.80	−0.10	–	–	–	–	–	–	0.42**
8. Item 8	1,171	1.59	0.91	1.53	1.73	–	–	–	–	–	–	–

**p < 0.01.

As Table 3 shows, not all the items included in the EMDS presented symmetric distribution. Indeed, the items Skewness indexes range from 0.80 to 2.65, while the Kurtosis indexes range from −0.10 to 6.75. All the items included in the scale are correlated with each other, but not too strongly.

### Factorial Structure of the Ethnic Moral Disengagement Scale and Its Discriminant Validity

**FIGURE 2:**
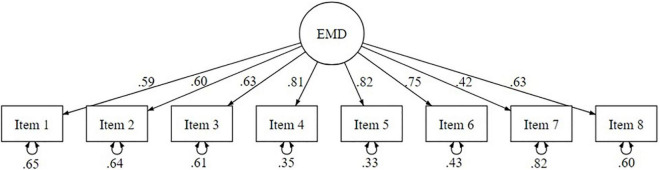
Graphical representation of the Ethnic Moral Disengagement monofactorial model (study 2).

STEP 1—CFA of the EMD scale—Our data were missing completely at random as indicated by the non-significant Little’s (1998) MCAR test, χ2(10) = 12, *p* = 0.284. Thus, after the estimation of missing data, we proceed to testing a monofactorial measurement model (CFA). All CFA model fit indices were satisfactory except for Chi Squared p, which is especially sensitive to sample size [χ2(20) = 68.84, *p* = 0.000]. Specifically, RMSEA, SRMR, and CFI had optimal values (RMSEA = 0.046; SRMR = 0.031; TLI = 0.951; CFI = 0.965). Moreover, all factor loadings estimated for the monofactorial model varied from βItem7 = 0.42 to βItem5 = 0.82. After its reformulation, Item 7, which had not shown satisfactory saturation in the first study, adequately saturated the latent factor EMD (β = 0.42; SE = 0.090; *p* < 0.001). Using Cronbach’s alpha coefficients to calculate the scale’s internal consistency, we found that the EMD scale showed a decent reliability: α = 0.84, 95% CI (0.85–0.86). The standardized estimates are reported in Figure 2.

STEP 2- Ethnic EMD scale measurement invariance—In Table 4 are reported the model’s fit indices for the comparison from the less restricted model (A–Configural Invariance: all parameters that are freely estimated) to the more constrained one (F–Latent Mean Invariance).

**TABLE 4:** Tests results for measurement invariance of EMD scale across ethnicity (Italians *N* = 904; Students with an immigrant background *N* = 275).

EMD models		Compared model	χ^2^ (df)	RMSEA	ΔRMSEA	CFI	ΔCFI	AIC	ΔAIC	BIC	ΔBIC
A	Configural Invariance		180.02 (40)	0.068		0.969		19,707.83		19,950.02	
B	Metric Invariance	A	184.43 (47)	0.060	−0.008	0.972	0.003	19,698.24	−9.58	19,905.12	−44.91
C	Scalar Invariance	B	189.19 (54)	0.055	−0.005	0.972	0.000	19,688.99	−9.24	19,860.55	−44.56
D	Strict Invariance	C	248.35 (62)	0.057	0.002	0.965	−0.007	19,732.15	43.16	19,863.35	2.79
E	Variance Invariance	D	255.73 (63)	0.058	0.001	0.964	−0.001	19,737.53	5.37	19,863.68	0.33
F	Latent Mean Invariance	E	258.53 (64)	0.058	0.000	0.963	−0.001	19,738.34	0.81	19,859.44	−4.24

The initial model A, that assessed configural invariance (Model A), resulted in an acceptable fit, as well as the second model B, testing the full metric invariance (Model B). Given that Model B leads to an acceptable CFI and RMSEA change compared to the configural invariance model (Model A), Metric Invariance was confirmed. For the third step of the measurement invariance test, the full scalar invariant model (Model C) resulted in an acceptable fit. Since Model C not too worsens Model B fit indices, Scalar invariance across ethnic backgrounds was confirmed. The fourth and fifth steps also, respectively, testing full strict invariance (Model D) and the factor variance invariance (Model E), yielded acceptable fits. Given that adding restrictions to the models, CFI and RMSEA fit indices do not particularly change, both the full strict invariance and the variance invariance across ethnic backgrounds were confirmed. Finally, the last step of invariance, testing latent mean invariance (Model F) showed an acceptable fit without significantly changing Model E fit indices. We may conclude that also latent mean invariance was also confirmed across the ethnic backgrounds.

STEP 3- Discriminant Validity—Table 5 shows the Pearson’s r correlation coefficients between EMD and Ethnic Bullying and between EMD and Ethnic Cyberbullying. EMD results significantly and positively correlated with both behaviors (Ethnic Bullying *r* = 0.160; *p* < 0.001; Ethnic Cyberbullying *r* = 0.185; *p* < 0.001).

**TABLE 5:** Correlations between EMD and ethnic bullying and ethnic cyberbullying.

	*N*	Mean (SD)	2.	3.
1. EMD	1,170	2.41 (0.34)	0.160*	0.185*
2. Ethnic Bullying	1,227	1.40 (0.11)		
3. Ethnic Cyberbullying	1,227	1.40 (0.10)		

*p < 0.001.

## General Discussion

Explanations for reprehensible conduct may reside in specific cognitive processes, which have been referred to as MD mechanisms, and which explain why common people are able to engage in unethical conduct, without experiencing apparent guilt (Bandura, 1991). The use of MD has been documented in several contexts, and it has been highlighted that it plays an important role in antisocial and aggressive behavior (Bandura et al., 1996, 2001; Menesini et al., 2003; Osofsky et al., 2005; Paciello et al., 2008; Fida et al., 2015). Despite its well-known importance for explaining aggressive conduct, such as bullying and cyberbullying (Gini et al., 2014; Lo Cricchio et al., 2021), the understanding of how MD operates within intercultural contexts remains at an early phase. Since MD mechanisms may be related to specific contextual characteristics, such as the ethnic or cultural origin of the potential victims, the investigation of EMD can be fundamental to prevent intolerance and discriminatory behaviors. However, to our knowledge, not much research has considered and measured the role of MD in the specific context of ethnic victims of bullying and cyberbullying episodes.

The aim of the present study was to develop a short, reliable, and valid scale for adolescents to assess MD in the case of ethnic minority potential victims. The conceptual referent theory of MD proposed by Bandura (1991) guided the development of the EMD scale, together with the use of some items concerning general MD. Each MD process was represented with one item. The initial pool of items was reviewed by an expert with research expertise related to the fields of ethnicity and MD. This guaranteed that items adequately represented the mechanisms they are planned to measure, and they were clearly phrased, brief and unequivocal. The scale was adjusted on the basis of this feedback, resulting in a final set of 8-items on a scale that was administered in two studies. In particular, study 1 was aimed at testing the initial hypothesized one-factor structure of the scale. However, study findings revealed some limitations of the scale deriving from the adequacy of Item 7 for measuring the latent factor of EMD. Therefore, we reformulated this item and administered this second version of the EMD scale in study 2 to test its factorial structure, internal consistency, and ethnic measurement invariance.

The results confirmed a one-factor model of EMD fitting the data well, with all fit indices being acceptable, the scale being internally consistent and reliable, and all items loading highly and signed onto the factor. These findings supported the presence of a single EMD factor, indicating that all mechanisms of MD are part of one general construct. This is consistent with Bandura’s (1991) theorizing that the MD maneuvers are different methods of accomplishing the same task: to disengage moral limitations from harmful behavior and decrease guilt for such conducts. In addition, these results are in line with previous studies, such as that of Boardley and Kavussanu (2008), which found that even though the items of some scales describe different mechanisms, there is evidence for a one-dimensional structure of the MD processes. So, even when we consider specific ethnic aspects of MD, all items referring to the eight theoretical mechanisms can be perceived as components of a unique common dimension that makes people inclined to use mechanisms of MD in interactions with people with a different ethnic background. Additionally, this study provides evidence of the internal consistency of the scale, which showed to be good, confirming the conceptual sense of the factor.

The invariance of the model across Italian adolescents and adolescents of different ethnicities/cultures of origin (i.e., having at least one parent born abroad) was supported through the examination of unconstrained and constrained models in the second sample of Study 2. In particular, we tested invariance constraining the latent factor means to be equal across the two groups. Results indicated that the scale works in the same way with students with or without immigrant background (i.e., majority vs minority), strengthening our confidence regarding the factorial integrity of the scale.

In order to assess the discriminant validity of the measure, we examined the links between the EMD factor and ethnic behavior of bullying, and cyberbullying. Results were in the expected direction. EMD showed to be positively associated with bullying peers of a different ethnicity or culture of origin. Similarly, a positive correlation was found between ethnic MD and online bullying of ethnic victims. Generally, these findings are in line with previous research, in which general MD has been positively associated with higher risk of engagement in ethnic victimization (Bayram Özdemir et al., 2020) and online racist form of harassment (Faulkner and Bliuc, 2016). However, it is important to note that correlations in our study were lower than expected. These results seem in line with those of Caravita et al. (2019), who found a lower level of specific MD when the victim is an immigrant peer rather than when he/she is a member of the autochthonous group. It is possible that the higher likelihood that immigrant people have of being victims of bullying episodes over time, both in cyberspace and in real life, has increased young people’s perception of ethnic bullying as more normative, and consequently, this has reduced individual’s need to justify (using MD maneuvers) these types of misconducts. Despite this, the ability of the EMD scale to be linked to these theoretically related constructs supports its usefulness in future research, in which ethnic MD’s role in ethnic bullying may be studied in association with other contextual and situational factors, such as the normativity of ethnic bullying behaviors.

One of the major benefits of the EMD scale is its brevity: the scale has both pragmatic power as well as sound psychometric properties. In light of these findings, the EMD scale appears to be a useful tool for those interested in measuring MD and predicting the occurrence of unethical or wrong behaviors toward victims belonging to minority groups.

### Study Limitations, Future Directions, and Conclusion

Some limitations should be noted. First, the items were developed to be used with adolescent samples, therefore the measure is appropriate for this specific demographic. However, further research into the psychometric properties of the EMD scale with more diverse age populations is encouraged. Secondly, both samples of the studies were recruited from schools in Italy. Keeping in view the scope of this study, the samples were adequate. However, for future research it would be beneficial to include samples from other countries so as to increase its generalizability and external validity. Third, the correlational design did not permit us to examine the longitudinal trajectories of the EMD scores. Future studies may pursue the aim of evaluating the stability of the measure over time. Last, we are aware that, despite the items of the EMD scale are derived from existing and validated measures, and are in line with the aim of measuring MD processes, there is the risk of legitimizing and/or reinforcing some prejudices toward immigrants. In light of these ethical concerns, our recommendation is to administer the scale together with others, which could highlight opposite attitudes and behaviors, such as tolerance toward diversity. Additionally, as previously stated, the scale has been developed in the Italian context, so it suits the language use of Italian adolescents. However, researchers from other countries, before using the scale, need to be aware of cultural and language differences and peculiarities in how adolescents talk in their everyday school contexts. As a consequence, in fact, there might be the need to adjust some of the items of the scale to better adapt to their specific ethical standards and beliefs.

Notwithstanding these limitations, the obtained findings start to shed light on the intricate aspects of ethnic MD as well as indicate that the EMD scale has considerable promise to be considered a useful measure to assess the related process in order to identify and prevent discriminative forms of aggression. It is easy to administer and it might attract a wide range of scientists, teachers, and educators who could take advantage from employing such a measure, which shows a balance between shortness and psychometric demandingness. In conclusion, we deem that future investigations on the adolescents’ ethnic MD are necessary and the EMD scale can be particularly helpful in this research.

## Data Availability Statement

The raw data supporting the conclusions of this article will be made available by the authors, without undue reservation.

## Ethics Statement

The studies involving human participants were reviewed and approved by Ethics Committee of the University of Florence. Written informed consent to participate in this study was provided by the participants’ legal guardian/next of kin.

## Author Contributions

BP, ML, and EM contributed to the conception and design of the study. FS organized the database and performed the statistical analysis. ML and FS wrote the first draft of the manuscript. BP, EM, and MP contributed to the manuscript revision. All authors read and approved the submitted version of the manuscript.

## Conflict of Interest

The authors declare that the research was conducted in the absence of any commercial or financial relationships that could be construed as a potential conflict of interest.

## Publisher’s Note

All claims expressed in this article are solely those of the authors and do not necessarily represent those of their affiliated organizations, or those of the publisher, the editors and the reviewers. Any product that may be evaluated in this article, or claim that may be made by its manufacturer, is not guaranteed or endorsed by the publisher.
